# The Effect of Cetylpyridinium Chloride Compared to Chlorhexidine Mouthwash on Scores of Plaque and Gingivitis: A Systematic Review and Meta‐Analyses

**DOI:** 10.1111/idh.12916

**Published:** 2025-06-18

**Authors:** Emmy Rowan Windhorst, Maud Joosstens, Eveline van der Sluijs, Dagmar Else Slot

**Affiliations:** ^1^ Department of Periodontology Academic Centre for Dentistry Amsterdam (ACTA), University of Amsterdam and Vrije Universiteit Amsterdam Amsterdam the Netherlands

**Keywords:** CHX, CPC, gingivitis, mouthrinse, mouthwash, plaque

## Abstract

**Aim:**

To evaluate the effectiveness of cetylpyridinium chloride (CPC) and chlorhexidine (CHX) mouthwashes (MW) on plaque and gingivitis scores for patients with gingivitis, in brushing as well as non‐brushing situations.

**Methods:**

A comprehensive search of MEDLINE‐PubMed and Cochrane‐CENTRAL was conducted to identify clinical and randomised controlled trials comparing CPC and CHX mouthwashes on plaque and gingivitis scores. The staining index was evaluated as a secondary outcome. In addition, the risk of bias was assessed. The data was summarised using a descriptive approach, and whenever possible, a meta‐analysis was conducted. The results for brushing and non‐brushing studies were presented separately. Grading was applied using the GRADE approach to rate the certainty of evidence.

**Results:**

The search resulted in 424 unique papers, from which 14 full‐text papers providing 18 comparisons were selected. Different concentrations of CPC‐MW (0.1%, 0.075%, 0.05%) and CHX‐MW (0.2%, 0.12%) were used. The risk of bias was estimated to be low, moderate or high for each study. A meta‐analysis for non‐brushing models showed a significant favour for CHX‐MW in plaque index scores (0.55 [95% CI: 0.19; 0.91], *p* = 0.003). For brushing, no significant differences were found between CPC‐MW and CHX‐MW. The descriptive analysis supports these findings. CHX‐MW tends to stain more than CPC‐MW.

**Conclusion:**

There is moderate certainty for a small statistically significant favourable effect of CHX‐MW over CPC‐MW for plaque control in non‐brushing situations, but no difference between them for plaque and gingivitis prevention in brushing situations.

## 1 Introduction

Periodontal diseases and dental caries are the most common diseases of humans and the main cause of tooth loss. Both diseases can therefore have negative impacts upon self‐esteem and quality of life [1]. The main etiological factor in caries and periodontal disease is the dental biofilm that forms and remains on tooth surfaces. Prevention should be based on approaches that counteract the dental plaque [2]. Therefore, the daily removal of dental plaque is required. Daily brushing and interdental cleaning are the most recommended mechanical oral hygiene procedures for oral self‐care. However, brushing and interdental cleaning are often insufficiently effective because of the poor compliance and inadequate skills of patients. For example, a systematic review evaluating over 10,000 subjects revealed that brushing removed on average only 42% of baseline plaque [3]. Therefore, the adjunctive use of chemical plaque control agents can be an effective complement to brushing for reducing plaque accumulation [4].

Chlorhexidine (CHX; 1,6‐bis(4‐chloro‐phenylbiguanido)hexane) is a cationic bis‐biguanide that is active against gram‐positive and gram‐negative organisms, facultative anaerobes, aerobes and yeasts [5]. The use of CHX mouthwash (MW) is well established in dental practice, has a robust scientific basis, and is considered the therapy of choice for those based on a chemical agent. There is strong evidence that using CHX‐MW as an adjunct to mechanical oral hygiene procedures reduces dental plaque and gingivitis scores [6, 7]. Even CHX‐MW used alone without mechanical oral hygiene procedures significantly reduces plaque accumulation [8, 9]. CHX‐MW is therefore widely used as the reference standard in research trials on MWs for daily use. However, the use of CHX‐MW by itself is restricted due to side effects that reduce patient compliance, such as burning sensation, taste alteration and extrinsic staining [6]. Prevailing evidence suggests that staining arises from the interaction between anionic dietary chromogens and adsorbed chlorhexidine cations [10, 11, 12]. These adverse effects make CHX‐MW a poor support of mechanical oral hygiene in the long term. Thus, there is a need for alternative MWs.

A frequently suggested alternative is cetylpyridinium chloride (CPC; 1‐hexadecylpyridinium chloride). CPC is a cationic surface‐active agent with a broad antimicrobial spectrum that is particularly effective in killing gram‐positive bacteria and yeasts [5]. Evidence supports the use of CPC‐containing MWs as an adjunct to oral hygiene, which provides a small but statistically significant benefit in reducing plaque accumulation and gingival inflammation [13]. In the light of the present evidence for MWs, CPC‐MW is sometimes suggested as an alternative and evidence‐based supported choice for long‐term daily use [5, 14, 15, 16, 17, 18], although it may also cause tooth staining [5]. However, a systematic review of studies evaluating the effect of CPC‐MW [13] identified only one study reporting statistically significant tooth staining caused by CPC‐MW [19]. To our knowledge, no systematic review has compared CPC‐MW and CHX‐MW. Such a systematic review comparing clinical studies can be used for up‐to‐date, evidence‐based patient advice and also as a reference in clinical practice guidelines.

The aim of this systematic review is to evaluate the effectiveness of CPC‐MW compared to CHX‐MW on plaque and gingivitis severity in patients with gingivitis, in brushing as well as in non‐brushing situations. In addition, tooth staining caused by these MWs is evaluated.

## 2 Materials and Methods

### 2.1 Review Question

What is the effect of CPC‐MW compared to CHX‐MW on scores of plaque and gingivitis in adult patients, both in brushing and non‐brushing situations?

### 2.2 Protocol and Guidelines

This systematic review and meta‐analysis were conducted and described following the guidelines outlined in the Cochrane Handbook for Systematic Reviews of Intervention and the Transparent Reporting of Systematic Reviews and Meta‐analyses (PRISMA statement) [20]. The protocol for this study was registered with both the International Prospective Register of Systematic Reviews (CRD42023444797) and the institutional review board of the Academic Centre for Dentistry Amsterdam (ACTA; ID: 2023‐85840) (Appendix S1). The crucial steps in the review process, including screening and selection for eligibility, quality assessment and estimating the risk of bias, data extraction and grading the body of evidence, were conducted using predefined procedures and forms independently by two reviewers (ERW and MJS). Disagreements were resolved by consensus, or if disagreement persisted, the judgement of a third reviewer (DES) or fourth reviewer (EvdS) was decisive.

### 2.3 Search Strategy

A structured search strategy was designed to retrieve all relevant published scientific studies from peer‐reviewed sources that evaluated the efficacy of CPC‐MW and CHX‐MW on plaque and gingivitis scores. The National Library of Medicine, Washington, D.C. (MEDLINE‐PubMed) and the Cochrane Central Register of Controlled Trials (CENTRAL) were searched from their inception up to May 2023 for appropriate papers that answered the question of interest. Furthermore, the reference lists of the included studies were manually searched to identify any additional potentially relevant studies. For specific information regarding the search terms employed, see Table 1.

**TABLE 1:** Search terms used for the search strategy.

{[intervention] AND [comparison]} {[<intervention **cetylpyridinium** > Textwords: (cetyl pyridinium) OR (cetylpyridinium) OR (CPC) OR (cetylpyridinium chloride) MesH: cetyl pyridinium] AND [<comparison **chlorhexidine** > Textwords: (chlorhexidine) OR (chlorhexidine di‐gluconate) OR (chlorhexidine gluconate) OR (zinc‐chlorhexidine) OR (chlorhexidine glucona te lidocaine hydrochloride) OR CHX OR (CHX formulations) OR (chlorhexidine phosphanilate) OR (chlorhexidine di‐actetate) MesH: chlorhexidine]}

### 2.4 Screening and Selection

The studies obtained from the searches were screened and selected. The software program Rayyan (https://www.rayyan.ai), an AI‐powered tool for systematic reviews, was utilised for article selection. The selection of relevance was a step‐by‐step process. Rayyan was used to identify and remove duplicates, which were subsequently sorted manually. The initial selection for relevance was based on titles and abstracts. Studies without abstracts were excluded at this stage. The second step in the selection for relevance was based on full‐text retrieval. No limitations were placed on the language or date of publication in the electronic searches of the databases.

The inclusion criteria were as follows:

- Randomised controlled clinical trials (RCTs) or controlled clinical trials (CCTs)
- Studies evaluating the effect of CPC‐MW and CHX‐MW
- Studies with a brushing or non‐brushing model
- Conducted on humans:
  ○ ≥ 18 years of age
  ○ In good general health (without systemic disorder)
- Minimum of 20 teeth
- For non‐brushing studies
  ○ Parameters of interest:
    ▪ Plaque index scores assessed according to one of the following most commonly used plaque indices or their modifications:
      ▫ Quigley and Hein plaque index (Q&HPI) [21] or the Turesky modification [22]
      ▫ Silness–Löe plaque index (S&LPI) [23]
      ▫ O'Leary plaque index [24]
- For brushing studies
  ○ Primary parameters of interest:
    ▪ Plaque index scores assessed according to one of the following most commonly used plaque indices or their modifications:
      ▫ Quigley and Hein plaque index (Q&HPI) [21] or the Turesky modification [22]
      ▫ Silness–Löe plaque index (S&LPI) [23]
      ▫ O'Leary plaque index [24]
    ▪ Gingivitis index scores assessed according to one of the following most commonly used gingivitis indices or their modifications:
      ▫ Silness–Löe gingival index (S&LPI) [23]
      ▫ Saxton & van der Ouderaa bleeding upon probing [25]
  ○ If available as a secondary parameter:
    ▪ Tooth discoloration assessed according to one of the following most commonly used stain indices or their modifications:
      ▫ Lobene stain index [26]
      ▫ Modified Lobene stain index [27]

The exclusion criteria were as follows:

- People hospitalised and/or with prosthodontics (prosthesis), orthodontics, dental implants.

### 2.5 Risk of Bias Assessment

Data were extracted from the papers that met all the inclusion criteria. All the included studies were assessed for risk of bias using the Cochrane RoB2 risk of bias tool to determine the influence of bias on the study results [28]. The items assessed included random sequence generation, allocation concealment, blinding of participants and personnel, blinding of outcome assessment, incomplete outcome data and selective reporting. Quality criteria were assigned a positive sign (+) if an informative description was present and the study design met the methodological criteria, a negative sign (−) if an informative description was present but the study design did not meet the criteria and a question mark (?) if information was missing or insufficient. Using the RoB2 tool, the risk of bias was judged to be ‘low,’ ‘moderate’ or ‘high.’

### 2.6 Data Extraction

The data obtained from the included studies was organised into evidence tables to evaluate the possibility of pooling for meta‐analysis. The tables encompass various aspects of clinical heterogeneity, such as study characteristics, assessment of study quality and the obtained results. Conflicts of interest (COIs) were classified into three types: donation of dental supplies, financial support from industry and whether the authors work in the industry.

Only the data relating to CPC‐MW and CHX‐MW was analysed; data related to other MWs or placebo groups was not extracted. Studies involving multiple variations of CPC or CHX MWs, such as different concentrations, were presented as separate comparisons within the evidence tables. The number of comparisons was summed. Among the papers included were brushing and non‐brushing studies. In studies following a non‐brushing model, participants were instructed to rinse with the MWs in the absence of other hygiene measures. The brushing and non‐brushing studies are presented separately.

To assess the plaque index and gingivitis index scores, a point estimate of the effect (mean) and an estimate of the variability (standard deviation; SD) were extracted. For studies that only provided the standard error (SE) of the mean, we calculated the SD using SE = SD/*n*, where *n* is the sample size when reported. To ensure an accurate estimate, data approximation from figures was avoided. In the case of missing data or undetermined information, we attempted to contact the first and/or corresponding author of the included publications to seek clarification or acquire additional data. If there was no response from the author, the data were excluded from the meta‐analysis.

### 2.7 Data Synthesis

As a summary, a descriptive data presentation was used for all studies regarding the comparison of CPC‐MW and CHX‐MW for plaque and gingivitis measurements. A meta‐analysis was performed on baseline, end and the change scores. Data from studies employing the same plaque index and gingivitis index was accumulated, and the weighted mean difference (WMD) was calculated. The study estimates were pooled using random‐effect models, and a fixed‐effect model was used when there were less than three studies included in the meta‐analysis. The meta‐analysis and confidence intervals for the effects of CPC‐MW and CHX‐MW were calculated using Review Manager (RevMan; version 5.4; for Windows, Copenhagen, Denmark: The Nordic Cochrane Centre, The Cochrane Collaboration, 2020). Details of the study design (non‐brushing and brushing) and the different concentrations of CPC and CHX used in the MWs were used to evaluate the heterogeneity of the outcomes. A subgroup analysis was performed when possible, and a sensitivity analysis was applied. A formal evaluation of publication bias was performed by visual inspection of the funnel plots, with a minimum of 10 comparisons, as proposed by Egger et al. [29].

### 2.8 Interpretation

A *p*‐value of less than or equal to 0.05 (≤ 0.05) was considered statistically significant. Little overlap of confidence intervals strongly indicated that statistical heterogeneity was present within the studies. If studies were deemed sufficiently similar in terms of the used methodology to allow comparison, a statistical examination of the heterogeneity was performed. The heterogeneity in the meta‐analysis was observed by eye‐balling and tested with the *I* ^2^ statistic [30]. As an approximate means of assessing the degree of inconsistency across studies, an *I* ^2^ statistic of 0%–40% was considered not potentially important, one of 30%–60% indicated moderate heterogeneity, one of 50%–90% represented substantial heterogeneity and a value of 75%–100% represented considerable heterogeneity [31]. Interpretation of the *I* ^2^ statistic can be found in Appendix S7.

### 2.9 Grading the Body of Evidence

The Grading of Recommendations Assessment, Development and Evaluation (GRADE) system was utilised to rank the evidence and determine certainty. Two reviewers (ERW and MJS) rated the certainty of the evidence as well as the strength and direction of the recommendations according to the risk of bias, consistency of results, directness of evidence, precision and publication bias and magnitude of the effect [32].

## 3 Results

### 3.1 Search and Selection Results

**FIGURE 1:**
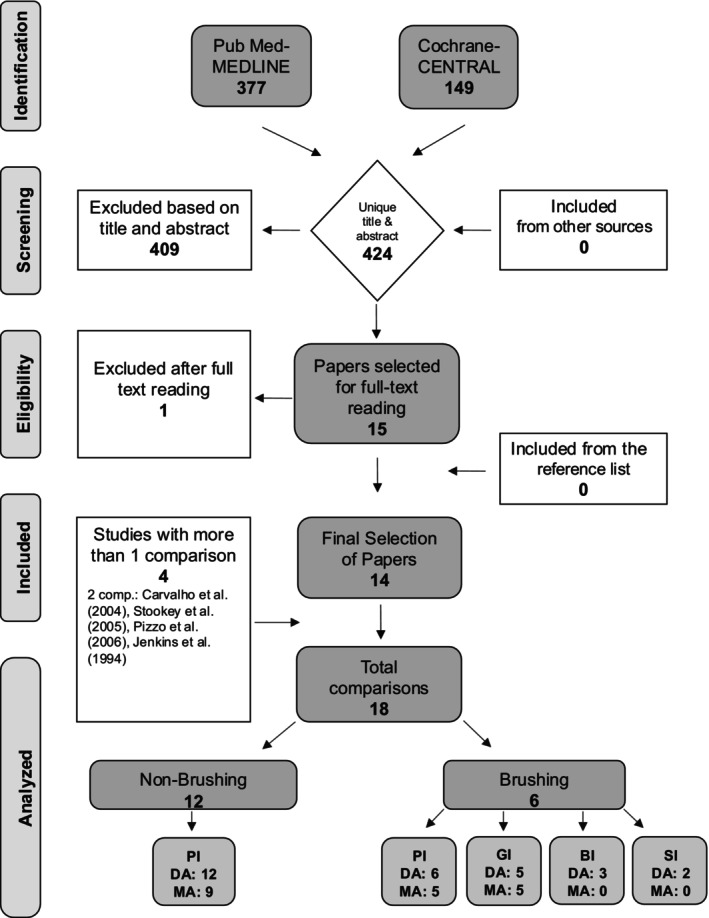
Flowchart of the search and selection process.

The search strategy identified 377 unique papers. Among the selection based on titles and abstracts, there were 11 COIs (< 3%) assessed by the reviewers (ERW and MJS). Based on the screening of the titles and abstracts, 15 papers were selected, of which the full text was read. One paper evaluated lozenges rather than CHX‐MWs and CPC‐MWs [33], and the other 14 papers were included in the systematic review. Among the included studies, four provided two comparisons involving different concentrations of CPC‐MW [34, 35, 36] and CHX‐MW [37]. As a result, 14 publications with 18 comparisons were included in this systematic review (for details, see Figure 1). In total, 12 comparisons were on non‐brushing, and in six comparisons, the MWs were used as an adjunct to brushing.

### 3.2 Risk of Bias Assessment

The estimated potential risk of bias of the included studies was assessed (for details, see Appendix S2). All the included studies were randomised controlled trials with a random generated sequence, but only one reported allocation concealment [38]. Five studies were single‐blinded, with either blinding of participants or blinding of the outcome assessment [35, 39, 40, 41, 42]. Five studies were double‐blinded [34, 38, 43, 44, 45], while the blinding status of four studies was uncertain [36, 37, 46, 47]. Three studies presented incomplete outcome data [35, 40, 45]. None of the studies presented other issues. The overall estimation of the potential risk of bias was high for 10 studies [35, 36, 37, 39, 40, 41, 42, 45, 46, 47] and moderate for three studies [34, 43, 44]. Only one study was graded as having a low risk of bias [38].

### 3.3 Study Characteristics and Heterogeneity Assessment

Information regarding the study design outline and characteristics is given in Table 2. The comparisons evaluated a total of 690 participants. All included studies were randomised controlled trials. Heterogeneity was observed in the studies regarding their study design, namely the use of a non‐brushing or brushing model, with a parallel or a crossover design. Different brands of CPC‐MW and CHX‐MW were used, with concentrations of 0.1%, 0.075% and 0.05% for CPC and 0.2% and 0.12% for CHX. The performed regimen exhibited heterogeneity regarding the rinsing regime, which was twice daily for 30 s [35, 42] or 60 s [34, 36, 37, 39, 40, 41, 44, 46, 47], and three studies did not specify the duration of rinsing [38, 43, 45]. The amount of MW used for rinsing was 10 mL [34, 37, 42, 46], 15 mL [35, 36, 39, 41] or 20 mL [44, 47].

**TABLE 2:** Characteristics of the included studies evaluating the effect of the use of CPC‐MW versus CHX‐MW. An overview of the studies processed for data extraction.

#. Authors (year), type of brushing model	Study design, duration, blinding	No. of participants baseline (end), gender, mean age, age range in years	Groups (brand)	Regimen: use and instruction	Funding	Conclusions of the original authors
I. Binney et al. (1992) [46], non‐brushing	RCT Single blind Crossover 4 days 3 days w.o.	18 (18) ♀: 10 ♂: 8 Mean age: ? Age range: 20–29	I: 0.05% CPC‐MW (Reach) C: 0.20% CHX‐MW (Corsodyl)	Day 1 professional prophylaxis Rinse 10 mL for 60 s 2×/day Suspend any other oral hygiene form	?	This study supports the plaque inhibitory properties of CHX and failed to support potential benefit to plaque inhibition of a CPC rinse
II. Jenkins et al. (1994) [34], non‐brushing	RCT Double blind Crossover 4 days 2.5 days w.o.	20 (18) ♀: 11 ♂: 9 Mean age: 26 Age range: 21–37	I1: 0.10% CPC‐MW (?) I2: 0.05% CPC‐MW (?) C1: 0.05% CHX‐MW (?)	Day 1 professional prophylaxis Rinse 10 mL for 60 s 2×/day Suspend any other oral hygiene form	SmithKline Beecham	Plaque inhibition with 0.1% CPC was significantly greater than 0.5% CHX. 0.05% CPC and 0.05% CHX were similar in efficacy
III. Renton‐Harper et al. (1996) [39], non‐brushing	RCT Single blind Crossover 4 days 2.5 days w.o.	20 (19) ♀: ? ♂: ? Mean age: ? Age range: 20‐35	I: 0.05% CPC‐MW (Oral‐B) C: 0.12% CHX‐MW (Procter & Gamble)	Day 1 professional prophylaxis Rinse 15 mL for 60 s 2×/day Suspend any other oral hygiene form	Oral‐B Laboratories, Redwood city, CA	CPC provided plaque inhibitory effects intermediate compared to CHX
IV. Moran et al. (2000) [41], non‐brushing	RCT Single blind Crossover 4 days 10 days w.o.	20 (20) ♀: ? ♂: ? Mean age: ? Age range: ?	I: 0.05% CPC‐MW (Scope) C: 0.12% CHX‐MW (Peridex)	Day 1 professional prophylaxis Rinse 15 mL for 60 s 2×/day Suspend any other oral hygiene form	?	CHX was found most effective, followed by CPC
V. Yates et al. (2002) [40], non‐brushing	RCT Single blind Parallel 5 weeks	35 (2) ◊ ♀: 43 ♂: 34 Mean age: 23 ◊ Age range: 19–38	I: 0.05% CPC‐MW (Sensodyne) C: 0.20% CHX‐MW (Corsodyl)	Rinse 10 mL for 60 s 2×/day Suspend any other oral hygiene form	?	CHX was highly significant more effective than CPC. The method achieved the expected result of differentiating between the CHX and the other rinses
VI. Carvalho et al. (2004) [36], non‐brushing	RCT Double blind Crossover 4 days 15 days w.o.	12 (12) ♀: 5 ♂: 7 Mean age: ? Age range: 19–23	I: 0.05% CPC‐MW (Cepacol) C2: 0.2% CHX‐MW (?) C1: 0.12% CHX‐MW (Periogard)	Day 1 professional prophylaxis Rinse 15 mL for 60 s 2×/day Suspend any other oral hygiene form	FAPESP	0.12% CHX and 0.2% CHX were significantly more effective in plaque inhibition than 0.05% CPC mouthwash
VII. Stookey et al. (2005) [35], brushing	RCT Double blind Parallel 6‐month	249 (212) ◊ ♀: 96 ◊ ♂: 153 ◊ Mean age: ? Age range: 18–66	I1: 0.10% CPC‐MW (?) I2: 0.075% CPC‐MW (?) C: 0.12% CHX‐MW (?)	Rinse 15 mL for 30 s Brushing manual with 0.243% NaF 2×/day	?	0.075% and 0.10% CPC shown statistically significant antiplaque and antigingivitis benefit over 6 months use
VIII. Pizzo et al. (2006) [37], non‐brushing	RCT Single blind Crossover 4 days 10 days w.o.	15 (15) ♀: 7 ♂: 8 Mean age: 23.45 SD age: 2.03 Age range: 22–27	I: 0.05% CPC‐MW (Iodosan) C2: 0.2% CHX‐MW (Corsodyl) C1: 0.12% CHX‐MW (Eburos)	Day 1 professional prophylaxis Rinse 10 mL for 60 s 2×/day Suspend any other oral hygiene form	?	Plaque scores were significantly lower with the CHX mouthrinses than with CPC mouthrinse
IX. Rahman et al. (2014) [43], non‐brushing	RCT Double blind Crossover 5 days 2 weeks w.o.	20 (20) ♀: 16 ♂: 4 Mean age: 22.55 SD age: 1.79 Age range: ?	I: 0.05% CPC‐MW (Aquafresh) C: 0.12% CHX‐MW (Oro‐Clens)	Rinse ? mL for ? s 2×/day Suspend any other oral hygiene form	University of Sharjah	0.05% CPC was found as efficient as CHX in dental plaque reduction
X. Junior et al. (2015) [42], brushing	RCT Single blind Parallel 3 weeks	20 (20) ◊ ♀: ? ♂: ? Mean age: 33.9 ◊ Age range: ?	I: 0.05% CPC‐MW (Sanofi) C: 0.12% CHX‐MW (Colgate)	Brushing 2 min Rinse 10 mL for 30 s 2×/day	?	The mouthwash containing CHX was the most effective controlling plaque, followed by CPC
XI. Tarlattinia et al. (2018) [47], non‐brushing	RCT Single blind Crossover 3 days 2 weeks w.o.	16 (16) ♀: 8 ♂: 8 Mean age: 20 SD age: ? Age range: 19–21	I: 0.075% CPC‐MW (Colgate) C: 0.2% CHX‐MW (Curasept)	Day 1 professional prophylaxis Rinse 20 mL for 60 s 2×/day Suspend any other oral hygiene form	?	The overall mean plaque index was lower with the use of CPC‐MW than with the use of CHX‐MW
XII. Miley et al. (2019) [45], brushing	RCT Double blind Parallel 6 weeks	49 ◊ (?) ♀: ? ♂: ? Mean age: ? Age range: ?	I: 0.05% CPC‐MW (Smarthmouth clinical DDS) C: 0.12% CHX‐MW (?)	Day 1 dental professional prophylaxis Rinse ? mL for ? s 2×/day In addition to MW, provided with dental floss (?) and ADA‐ accepted toothbrushes (?)	Triumph Farmaceuticals	Both CPC and CHX shown to improve GI, BS and PI scores
XIII. Tadakamadla et al. (2020) [38], brushing	RCT Double blind Parallel 21 days	50 (50) ◊ ♀: 38 ◊ ♂: 12 ◊ Mean age: 20.6 SD age: 0.8 Age range: 20–23	I: 0.05% CPC‐MW (G.&G.V. Gengyve) C: 0.12% CHX‐MW (Rexidin)	Day 1 professional prophylaxis Rinsing ? mL for ? s 2×/day In addition to MW, provided with fluoridated toothpaste (Colgate Strong teeth) and toothbrushes (Colgate Sensitive Ultra Soft)	CDR Pharma, Milan, Italy, that provided the G.&G.V. Gengyve mouthwash	CPC and CHX had similar effectiveness in preventing plaque accumulation, while no differences were observed for preventing gingivitis
XIV. Oo et al. (2023) [44], brushing	RCT Double blind Parallel 6 weeks	146 (146) ◊ ♀: ? ♂: ? Mean age: 22.9 SD age: 1.47 Age range: 20–25	I: 0.05% CPC‐MW (Colgate) C: 0.12% CHX‐MW (Colgate)	Brushing with patients' normal technique with fluoride toothpaste Rinse 20 mL for 60 s 2×/day	University Sains Malaysia Short Term Grant	When used twice daily, the effectiveness of CPC was comparable with CHX

*Note:* ♀ = female; ♂ = male; ◊ = calculated by the authors of this review based on the presented data in the selected paper; ? = not reported/unknown.

Abbreviations: C = control; CHX = chlorhexidine; CPC = cetylpyridinium chloride; F = fluoride; I = intervention; MW = mouthwash; NaF = sodium fluoride; OE = essential oils; RCT = randomised controlled trial; w.o = washout period for crossover design.

### 3.4 Conflict of Interest and Funding

Four included papers reported no COI [42, 43, 44, 47], one paper reported that the MWs used were donated [38], and four papers reported financial support from the dental industry [34, 36, 39, 45]. Seven papers did not provide any explicit disclosure of a COI [35, 37, 40, 41, 42, 46, 47].

### 3.5 Study Outcome Results

#### 3.5.1 Data

##### 3.5.1.1 Data Extraction

Appendix S3a–d presents the results of extracting plaque index scores (Appendix S3a) separated for non‐brushing and brushing studies. In addition, for the brushing studies, the reported bleeding index scores (Appendix S3b), gingival index scores (Appendix S3c) and staining index (Appendix S3d) are presented. Tables 3a and 3b provide a descriptive summary of the statistical significance for CPC‐MW compared to CHX‐MW based on the 18 comparisons for the four outcomes of interest. Tables 3a and 3b present the descriptive summaries for the non‐brushing and brushing studies, respectively.

**TABLE 3a:** Summary of the descriptive analysis for non‐brushing study models.

Study no.	Intervention	PS	Comparison
II. Jenkins et al. (1994) [34]	0.10% CPC‐MW	+	0.05% CHX‐MW
	0.05% CPC‐MW	0	
III. Renton‐Harper et al. (1996) [39]	0.05% CPC‐MW	−	0.12% CHX‐MW
IV. Moran et al. (2000) [41]	0.05% CPC‐MW	?	0.12% CHX‐MW
IX. Rahman et al. (2014) [43]	0.05% CPC‐MW	0	0.12% CHX‐MW
VI. Carvalho et al. (2004) [36]	0.05% CPC‐MW	−	0.12% CHX‐MW
		−	0.20% CHX‐MW
VIII. Pizzo et al. (2006) [37]	0.05% CPC‐MW	−	0.12% CHX‐MW
		−	0.20% CHX‐MW
I. Binney et al. (1992) [46]	0.05% CPC‐MW	−	0.20% CHX‐MW
V. Yates et al. (2002) [40]	0.05% CPC‐MW	−	0.20% CHX‐MW
XI. Tarlattinia et al. (2018) [47]	0.075% CPC‐MW	0	0.20% CHX‐MW
Summary	CPC‐MW	7/11: − (63.6%) 3/11: 0 (27.3%) 1/11: + (9%)	CHX‐MW

*Note:* Green (+) = significant difference in favour of the cetylpyridinium chloride group; Red (−) = significant difference in favour of the chlorhexidine group; Orange (0) = no significant difference; ? = not reported/unknown.

Abbreviations: CHX = chlorhexidine; CPC = cetylpyridinium chloride; MW = mouthwash; PS = plaque score.

**TABLE 3b:** Summary of the descriptive analysis for brushing study models.

Study no.	Intervention	PS	GI	BS	SI	Comparison
X. Junior et al. (2015) [42]	0.05% CPC‐MW	?	□	□	□	0.12% CHX‐MW
IX. Miley et al. (2019) [45]	0.05% CPC‐MW	?	0	0	□	0.12% CHX‐MW
XI. Tadakamadla et al. (2020) [38]	0.05% CPC‐MW	0	0	□	0	0.12% CHX‐MW
XIV. Oo et al. (2023) [44]	0.05% CPC‐MW	0	0	□	+	0.12% CHX‐MW
VII. Stookey et al. (2005) [35]	0.075% CPC‐MW	?	?	?	□	0.12% CHX‐MW
	0.10% CPC‐MW	?	?	?	□	
Summary	CPC‐MW	2/2: 0 (100%)	3/3: 0 (100%)	1/1: 0 (100%)	1/2: 0 (50%) 1/2: + (50%)	CHX‐MW

*Note:* + = significant difference in favour of the cetylpyridinium chloride group; − = significant difference in favour of the chlorhexidine group; 0 = no significant difference; □ = no data available; ? = not reported/unknown.

Abbreviations: BS = bleeding score; CHX = chlorhexidine; CPC = cetylpyridinium chloride; GI = gingival index; MW = mouthwash; PS = plaque score; SI = staining score.

##### 3.5.1.2 Data Presentation

Tables 4a and 4b summarise the meta‐analysis results for the non‐brushing and brushing studies, respectively. Further subgroup analysis was performed for different concentrations of CPC‐MW and CHX‐MW. The possible comparisons were 0.05% CPC‐MW versus all CHX‐MW; all CPC‐MW versus 0.12% CHX‐MW; all CPC‐MW versus 0.2% CHX‐MW; 0.05% CPC‐MW versus 0.12% CHX‐MW; and 0.05% CPC‐MW versus 0.2% CHX‐MW. Appendices S4–S6 contain the original forest plots of the meta‐analyses. Testing for publication bias could not be performed because fewer than 10 comparisons or studies were included in the meta‐analysis.

**TABLE 4a:** Summary of the meta‐analysis of CPC‐MW compared to CHX‐MW for non‐brushing study model for plaque index scores.

Comparison	No. of included studies	No. of comparisons	Model	MD	Test overall		Test for heterogeneity		For details see the appendix
					95% CI	*p*	*I* ^2^ value (%)	*p*	
Overall CPC vs. CHX	7 [34, 37, 39, 41, 43, 46, 47]	9	Random	0.55	0.19; 0.91	**0.003**	93%	**< 0.0001**	S4a
Sub‐analysis 0.05% CPC vs. all CHX	7 [34, 37, 39, 41, 43, 46, 47]	8	Random	0.64	0.26; 1.02	**0.001**	93%	**< 0.0001**	S4b.1
Sub‐analysis All CPC vs. 0.12% CHX	6 [34, 37, 39, 41, 43, 47]	7	Random	0.38	−0.01; 0.76	0.06	92%	**< 0.0001**	S4b.2
Sub‐analysis All CPC vs. 0.2% CHX	2 [37, 46]	2	Fixed	0.80	0.61; 0.99	**< 0.0001**	95%	**< 0.0001**	S4b.3
Sub‐analysis 0.05% CPC vs. 0.12% CHX	5 [34, 37, 39, 41, 43]	5	Random	0.61	0.22; 1.01	**0.002**	88%	**< 0.0001**	S4b.4
Sub‐analysis 0.05% CPC vs. 0.2% CHX	2 [37, 46]	2	Fixed	0.80	0.61; 0.99	**< 0.0001**	95%	**< 0.0001**	S4b.5

*Note:* *p*‐values are presented in bold if *p* ≤ 0.05. For interpretation colours of the *I* ^2^, see Appendix S7.

Abbreviation: NA = not applicable.

**TABLE 4b:** Summary of the meta‐analysis of CPC‐MW compared to CHX‐MW for brushing study model for plaque index scores.

Comparison	Measurement moment	No. of included studies	No. of comparisons	Model	MD	Test overall		Test for heterogeneity		For details see the appendix
						95% CI	*p*	*I* ^2^ value (%)	*p*	
Overall	Baseline	4 [35, 38, 44, 45]	5	Random	0.01	−0.02; 0.05	0.47	25%	0.26	S5a.1
	End	4 [35, 38, 44, 45]	5	Random	0.04	−0.01; 0.10	0.11	74%	**0.004**	S5a.2
	Change scores	3 [38, 44, 45]	3	Random	0.03	−0.06; 0.13	0.51	12%	0.32	S5a.3
Sub‐analysis 0.05% CPC vs. 0.12% CHX	Baseline	3 [38, 44, 45]	3	Random	0.01	−0.03; 0.05	0.71	39%	0.19	S5b.1
	End	3 [35, 38, 44, 45]	3	Random	0.02	−0.02; 0.05	0.35	64%	0.06	S5b.2

*Note:* *p*‐values are presented bold if *p* < 0.05. Interpretation of the colour shades are described in Appendix S7. Reflecting the *I* ^2^ value interpetation: Green = potential not important; Yellow = moderate; Orange = substantial; and Red = considerable.

#### 3.5.2 Non‐Brushing Studies

##### 3.5.2.1 Descriptive Analysis

For non‐brushing studies, a total of 12 comparisons of plaque scores between CPC‐MW and CHX‐MW were found. Out of 12 comparisons, seven (63.6%) found that CHX‐MW was significantly more effective than CPC‐MW. The only comparison of 0.10% CPC‐MW to 0.05% CHX‐MW concluded a significant result in favour of CPC‐MW (for details, see Table 3a).

##### 3.5.2.2 Meta‐Analysis

For the non‐brushing comparisons, the meta‐analysis performed on the end data of plaque index scores is summarised in Table 4a, and the original forest plots are presented in Appendix S4a,b. The overall meta‐analysis revealed an effect in favour of CHX‐MW (0.55 [95% CI: 0.19; 0.91], *p* = 0.003), where the heterogeneity (*I* ^2^ = 93%) was considerable. In total, all five subgroup comparisons were performed, and considerable heterogeneity (88%–95%) was observed. Four of the five comparisons concluded in favour of CHX‐MW (*p* < 0.002). The only comparison that did not indicate a significant difference was for all concentrations of CPC‐MW compared to 0.12% CHX‐MW.

#### 3.5.3 Brushing Studies

##### 3.5.3.1 Descriptive Analysis

The brushing studies included six comparisons. All evaluated the primary outcome plaque scores, but not all evaluated gingivitis and bleeding scores or the staining score. Moreover, not all comparisons and outcomes of interest of the studies (*N* = 8) presented statistical evaluations. For the plaque, gingivitis and bleeding scores, none of the presented comparisons indicated a statistically significant difference between CPC‐MW and CHX‐MW. No difference was also the case for one of the comparisons [38] on the staining scores. Another study found that CHX‐MW resulted in significantly more staining than CPC‐MW [44].

##### 3.5.3.2 Meta‐Analysis

For the brushing comparisons, the meta‐analysis could only be performed and summarised for the plaque index scores (Table 4b) and the gingival index scores (Table 4c). The original forest plots are presented in Appendices S5 and S6. The meta‐analysis on the plaque scores (Table 4b) was based on five comparisons for the baseline, end and change scores; none of which revealed a significant difference (*p* > 0.11). The heterogeneity for the baseline (25%) and change scores (12%) was considered not potentially important. For the end scores, substantial heterogeneity of 74% was observed. The subgroup analysis for the comparison of 0.05% CPC‐MW versus 0.12% CHX‐MW on baseline and end scores, neither of which revealed a significant difference (*p* > 0.35). The heterogeneity was considered to be moderate (39%) for the baseline scores and substantial for the end scores (64%). The same comparisons were performed for the gingival index scores (Table 4c), and no significant difference was found (*p* > 0.20). In the overall analysis, the heterogeneity was low for the baseline (0%) and change (0%) scores but considerable for the end score (55%). In the subgroup analysis of 0.05% CPC‐MW versus 0.12% CHX‐MW, moderate heterogeneity of 40% was observed for the baseline score, and negligible heterogeneity was observed for the end score (0%).

**TABLE 4c:** Summary of the meta‐analysis of CPC‐MW compared to CHX‐MW for brushing study model for gingival index scores.

Comparison	Measurement moment	No. of included studies	No. of comparisons	Model	MD	Test overall		Test for heterogeneity		For details see the appendix
						95% CI	*p*	*I* ^2^ value (%)	*p*	
Overall	Baseline	4 [35, 38, 44, 45]	5	Random	0.00	−0.02; 0.01	0.58	0%	0.49	S6a.1
	End	4 [35, 38, 44, 45]	5	Random	0.02	−0.01; 0.05	0.20	55%	0.06	S6a.2
	Change scores	3 [38, 44, 45]	3	Random	0.01	−0.03; 0.04	0.70	0%	0.54	S6a.3
Sub‐analysis 0.05% CPC vs. 0.12% CHX	Baseline	3 [38, 44, 45]	3	Random	−0.01	−0.04; 0.02	0.37	40%	0.19	S6b.1
	End	3 [38, 44, 45]	3	Random	0.00	−0.01; 0.01	0.99	0%	0.99	S6b.2

*Note:* *p*‐values are presented bold if *p* < 0.05. Interpretation of the colour shades are described in Appendix S7. Reflecting the *I* ^2^ value interpetation: Green = potential not important; Yellow = moderate; Orange = substantial; and Red = considerable.

### 3.6 Evidence Profile

Table 5 provides a summary of the various factors used to rate and assess the certainty for the quality of evidence and strength of recommendations according to GRADE [32]. There is moderate certainty for a small statistically significant favourable effect of CHX‐MW over CPC‐MW for plaque control in non‐brushing situations but no difference between them for plaque and gingivitis prevention in brushing situations.

**TABLE 5:** Summary of the findings on the body of the estimated evidence profile and appraisal of certainty and the strength of the recommendation regarding CPC‐MW versus CHX‐MW on measurements of plaque and gingivitis.

Determinants of the quality	Non‐brushing	Brushing
Study design	RCT	RCT
No. of studies (Figure 1, Table 2)	9	5
No. of comparisons (Figure 1)	12	6
No. of meta‐analysis (Tables 3a and 3b, Appendices S4 and S5)	9	5
Risk of bias (Appendix S2)	Moderate to high	Low to high
Consistency (Appendices S4 and S5)	Rather consistent	Rather consistent
Directness	Generalizable	Generalizable
Precision (Appendices S4 and S5)	Rather precise	Rather precise
Reporting bias	Possible	Possible
The magnitude of the effect	Small	None
Strength of the recommendation based on the quality and body of evidence	Moderate	Moderate
Overall recommendation	**There is moderate certainty for a small statistically significant favourable effect of CHX‐MW over CPC‐MW for plaque control in non‐brushing situations but no difference between them for plaque and gingivitis prevention in brushing situations.**	

## 4 Discussion

The aim of this systematic review was to summarise the available literature on the effect of CPC‐MW compared to that of CHX‐MW in terms of plaque and gingivitis, in brushing as well as in non‐brushing situations. Staining scores were evaluated as a secondary outcome of interest. The results in this systematic review obtained from both the descriptive analysis and the meta‐analyses were consistent. In the non‐brushing studies, the plaque index scores were statistically significant in favour of CHX‐MW with a moderate effect. Both CPC‐MW and CHX‐MW reduced plaque and gingivitis without a significant difference between them when used as adjuncts to brushing. There was no conclusive evidence for the staining score; staining is an important side effect that influences patient acceptance and compliance. However, as an overall result, CPC‐MW can serve as a suitable substitute for CHX‐MW for long‐term use. However, for indications where plaque control is the main focus, such as in post‐surgery wound healing, CHX‐MW remains the first choice.

### 4.1 Brushing vs. Non‐Brushing Study Designs

Among the included papers, both non‐brushing and brushing designs were used to estimate the effect of CPC‐MW compared to that of CHX‐MW. The non‐brushing studies estimated plaque regrowth, whereas the brushing studies estimated plaque reduction. From the evaluation of the non‐brushing studies, a significant effect was found in favour of CHX‐MW. A non‐brushing study allows the effect of the MW to be estimated without the influence of brushing [48], which is considered the most effective mechanical way to remove plaque [2, 50, 51]. Removing this influence provides a pure estimate of the effect, but it does not reflect the actual habitual use of the product by the patients [49]. Sometimes it is not possible to mechanically remove plaque by a toothbrush, and only chemical plaque removal is possible, such as in post‐surgery wound healing [52]. The American Dental Association (ADA) requires an evaluation of the efficacy and safety of a chemical agent by a long‐term study with a minimum duration of 6 months with an intermediate evaluation at 3 months before it issues a seal of acceptance for the agent [53]. This requirement also applies to CPC and CHX MWs. For brushing studies, the meta‐analysis was performed on plaque and gingivitis index scores, and no statistically significant difference was observed between CPC and CHX. Further long‐term studies could be performed using other measures of gingival inflammation, such as the bleeding score. This score reflects the results of a clinical evaluation in a regular clinic, as bleeding upon probing is part of regular periodontal assessment and diagnosis [54].

### 4.2 Essential Oils MW

Essential‐oil mouthwash (EO‐MW) is considered the first‐choice alternative to CHX‐MW with respect to gingivitis [8]. EO‐MW is found in an over‐the‐counter MW with a formulation consisting of two phenol‐related essential oils: thymol at 0.064% and eucalyptol at 0.092% [5]. It has been demonstrated that, compared to a standardised EO‐MW formulation, CHX‐MW resulted in better plaque scores. However, no long‐term significant difference with respect to the reduction of gingival inflammation was found. Furthermore, CHX‐MW caused considerably more side effects. Therefore, EO‐MW appears to be a viable alternative to CHX‐MW for reducing gingival inflammation [8].

A major drawback in the use of EO‐MW in its original composition is the high alcohol content (22%–27%). The alcohol is used as a preservative and as a solvent in the preparation. The safety of MWs containing alcohol has been discussed, studied and evaluated by systematic reviews. Three independent systematic reviews concluded that there is no sufficient evidence to suggest that MWs containing alcohol can influence oral cancer [9, 55, 56]. Therefore, it is important to note that current studies do not establish a clear causal relationship between alcohol MW use and oral cancer [9, 55, 56]. Nevertheless, alcohol‐free rinses are attracting increasing interest, particularly among groups that avoid alcohol for religious reasons.

### 4.3 Effects of Concentration and Rinse Duration

Different concentrations of CPC and CHX were used in the MWs in the examined studies in this review. A recent review indicates that CHX‐MWs are most recommended at concentrations of 0.12%–0.2% [57]. Differences in concentration may influence the effect of the MWs for the outcomes of interest. A significant benefit of using CPC‐MW was only observed when the highest concentration of CPC (0.1%) was compared to the lowest concentration of CHX (0.05%) [34]. The influence of the MW concentration was consistent with results from a previous systematic review that evaluated the effect of 0.12% and 0.2% CHX MWs on plaque and gingivitis [58]. It concluded that there was a small but statistically significant difference in the effect on plaque for the two CHX concentrations. However, it suggested that the clinical relevance of this difference is probably negligible. The evidence regarding gingival inflammation for these concentrations, however, is sparse. According to the limited available research, no difference between the two concentrations in terms of reducing gingivitis could be established [58]. The optimal dosage of CHX is commonly regarded as approximately 20 mg administered twice daily [59, 60]. According to manufacturer instructions, 15 mL of 0.12% CHX solution or 10 mL of 0.2% CHX is administered, giving similar doses of 18 and 20 mg CHX, respectively. A dose of 18 mg has been suggested as the minimum amount of CHX needed when incorporated in an MW [61, 62]. The included study with 0.05% CHX‐MW [34] used a rinsing volume of 10 mL, giving a dose of 5 mg per rinsing episode. This is only about 25% of the required amount needed for optimum of 18 mg CHX. Another clarification is that the significant effect in favour of 0.1% CPC when compared to the 0.05% CHX can be that the CPC is 0.1%, a rather high percentage. Although it can be considered as high, the formulation meets the ADA's safety limit for CPC concentration [13]. There was also heterogeneity in the volume (15–30 mL) and rinse duration (30–60 s) among the studies. A study that assessed the effect of different rinsing times observed no significant effect on plaque inhibition for rinse durations of 15, 30 and 60 s [63]. In contrast, a systematic review revealed a small significant difference between rinse times of 30 and 60 s for CHX for plaque scores [58]. However, rinsing for 30 s appears to be sufficient for all the surfaces of the dentition to come in contact with the MW [6], with all the studies in the present systematic review employing a rinsing time of at least 30 s.

### 4.4 Side Effects

Side effects of chemical MWs can affect patient compliance and motivation. Some of the studies included in this systematic review evaluated the side effects of both MWs as part of their protocol, in addition to their effects on plaque and gingivitis scores. Staining is the most commonly known side effect of CHX‐MW [6, 7]. Two of the included brushing studies investigated tooth staining as a secondary measurement of interest in patients using CPC‐MW and CHX‐MW [38, 44]. One found no significant difference [38], but stated that the tooth staining score was three times higher for CHX‐MW than for CPC‐MW, which raises questions about the interpretation of these results because a three times higher score is often statistically significant. The other study [44] revealed significantly lower tooth staining scores for the CPC‐MW group than for the CHX‐MW group. In addition, it found that taste alteration and numbness were more common among the patients using CHX‐MW [44]. Tooth staining caused by CPC‐MW has a similar dietary aetiology to that induced by CHX‐MW, but it appears to be less severe [13]. This may be due to the phenomenon of substantivity: CHX‐MW lasts longer in the mouth than CPC‐MW [5]. The duration of the therapeutic effect, as measured by residual salivary antibacterial activity, is up to 90 min for CPC‐MW, compared to 7 h for CHX‐MW [64]. Due to this substantivity, prolonged use can reduce bitter and salty taste sensations, potentially making patient compliance more challenging [5].

For the non‐brushing studies, only plaque measurements were included in the present systematic review. Non‐brushing studies traditionally have a short duration of 4–5 days [8], whereas side effects associated with CHX typically manifest in long‐term use over a period of at least 4 weeks [6]. However, two short‐term non‐brushing studies [37, 43] also evaluated tooth staining in general as being either present or absent. One [43] observed tooth staining in two cases and the other observed it in one case [37] within the CHX‐MW group, corresponding to 20% and 7% of the original study sample, respectively. Neither reported cases of tooth staining for CPC‐MW. After taking into account potential side effects, CPC‐MW could be preferable over CHX‐MW.

Side effects play a crucial role in chemical plaque control, but most of the included papers are timid in reporting them. According to the CONSORT Harms guidelines, the need to report both benefits and harms is essential in randomised controlled trials. Therefore, we recommend conducting new clinical trials with side effects as a secondary outcome [65].

### 4.5 Patient Preferences

Patient preferences, motivation and compliance play a crucial role in evidence‐based practice [66]. It is also important to incorporate the patient's judgement when making decisions about their treatment plan. In this review, only one of the studies [47] investigated patient preferences in addition to the clinical research‐oriented outcomes. Participants completed a questionnaire with a visual analogue scale to indicate their satisfaction with and perception of the MW that they used. The taste of CPC‐MW was significantly preferred by patients, was perceived to have a shorter aftertaste, and was found to be more convenient to use than CHX‐MW [47]. We recommend measuring patient‐oriented outcomes using a questionnaire in addition to traditional clinical outcomes to reflect the patient's viewpoint.

### 4.6 Bias Considerations

The findings of a systematic review strongly rely on the methodological quality, the validity and the risk of bias of the studies included in the meta‐analysis. The present systematic review performed meta‐analysis on the baseline, end and changes scores. The meta‐analysis on the baseline scores was done to be sure the randomisation procedures resulted in correct and comparable groups before the intervention. If only end scores were taken into account, the potential effect of non‐effect could be based on a difference at baseline. For the present systematic review, this applies in particular for the sub‐analysis in the brushing studies. In contrast, the meta‐analysis on the change scores incorporated differences at baseline, which were used for the overall analysis. Another potential aspect of bias is that now only data of overall index scores were taken into account; this can be considered a limitation. It could be valuable and recommended to analyse differences between proximal and free surfaces as a secondary analysis, based on individual patient‐level data. Secondary analysis allows for the opportunity to conduct meta‐analyses and explore new research questions or hypotheses that were not the primary focus of the original studies [67].

Among the studies included in this systematic review, 90% were judged as having a high or moderate risk of bias, with only one study identified as having a low risk of bias [38]. This finding compromises the validity of the meta‐analysis. The studies included were not rated as sufficiently robust to provide high confidence in the estimated effect. In addition, selection bias was evident in four of the studies [36, 37, 44, 45]. These studies utilised dental students as participants, who do not accurately represent the target population of this review [68]. We recommend conducting new clinical trials with a study design including a representative sample that ensures minimal influence of bias, which could add value to further assessments comparing the effectiveness of CPC‐MW and CHX‐MW.

### 4.7 Conflicts of Interest and Sources of Funding

Transparency among authors regarding the study development is very important for public trust in the scientific process. Potential for COIs exists when professional judgement concerning a primary interest may be influenced by a secondary interest, such as financial gain [69]. Other interests, such as personal relationships or rivalries, academic competition and intellectual beliefs, may also present COIs [69]. In this review, two types of COI were reported in the included papers: the donation of dental supplies and financial support from the dental industry. These COIs were reported in five of the 14 papers. Moreover, secondary interests, including financial gain, cannot be ruled out. The majority of studies in this review did not provide explicit disclosure of any COI [35, 37, 40, 41, 42, 46, 47], which may indicate a greater risk of COIs, potentially introducing bias in the effects observed in this review. Journals, editors and reviewers should focus on COIs and funding statements, in line with the current standards of the ICMJE guidelines [69]. On the other hand, it is difficult to obtain funding for studies on dental homecare devices through grants from public funding agencies or the not‐for‐profit sector. Such studies are almost entirely self‐funded or rely on commercial support.

## 5 Conclusion

Our analysis of the 14 studies comparing CHX‐MW to CPC‐MW in this meta‐analysis revealed that there is moderate certainty of a small statistically significant favourable effect of CHX‐MW over CPC‐MW for plaque control in non‐brushing situations but no difference between them for plaque and gingivitis prevention in brushing situations. We conclude that in brushing situations, CPC‐MW can serve as a substitute for CHX‐MW.

## 6 Clinical Relevance

### 6.1 Scientific Rationale

CHX‐MW is widely used as the reference standard MW, and CPC‐MW is sometimes suggested as an alternative.

### 6.2 Principal Findings

There is moderate certainty of a small statistically significant favourable effect of CHX‐MW over CPC‐MW in terms of plaque index scores in non‐brushing situations but no difference in the effect of CPC‐MW and CHX‐MW for plaque and gingivitis scores in brushing situations.

### 6.3 Practical Implications

For indications where plaque control is the main focus, such as post‐surgery wound healing, CHX‐MW remains the first choice. For long‐term use, CPC‐MW appears to be a reliable alternative to CHX‐MW with respect to measurements of plaque and gingival inflammation.

## Author Contributions

All authors gave their final approval and agreed to be held accountable for all aspects of the work, ensuring integrity and accuracy. Emmy Rowan Windhorst contributed to conception and design, analysis, interpretation and drafted the manuscript. Maud Joosstens contributed to conception and design, analysis, interpretation and critically revised the manuscript. Eveline van der Sluijs contributed to conception and design, interpretation and critically revised the manuscript. Dagmar Else Slot contributed to conception and design, analysis and interpretation and critically revised the manuscript.

## Disclosure

Slot and van der Sluijs have previously received either external advisor fees, lecturer fees or research grants from toothbrush manufacturers and brands. Those included Colgate, Dentaid, GABA, Lactona, Oral‐B, Procter & Gamble, Philips, Sara Lee, Sunstar, Waterpik and Unilever.

## Conflicts of Interest

The authors declare no conflicts of interest.

## Supporting information


Appendices S1–S7

## Data Availability

The data that support the findings of this study are available in the Supporting Information of this article.
