# Supplementary material for: The Effect of Cetylpyridinium Chloride Compared to Chlorhexidine Mouthwash on Scores of Plaque and Gingivitis: A Systematic Review and Meta‐Analyses
Source: Int J Dent Hyg. 2025 Jun 18;23(4):665–81. doi: 10.1111/idh.12916 (PMC12516004; doi:10.1111/idh.12916)
Supplement: Supplementary file 1 — Appendices S1–S7 [file IDH-23-665-s001.docx]

**The effect of cetylpyridinium chloride mouthwash compared to chlorhexidine mouthwash on scores of plaque and gingivitis**

***=a systematic review and meta-analyses=***

**Emmy R Windhorst** (https://orcid.org/0009-0000-1137-0136)

**Maud Joosstens** (https://orcid.org/0009-0003-0180-6495)

**Eveline van der Sluijs** (https://orcid.org/0000-0003-1269-1710)

**Dagmar Else Slot** (https://orcid.org/0000-0001-7234-0037)

**Appendices**

Appendix 1. Ethical approval of the ACTA institute

Appendix 2. Risk of Bias assessment

Appendix 3. Data extraction tables

Appendix 4. Forest plot meta-analysis for non-brushing studies

Appendix 5-6. Forest plot meta-analysis for brushing studies

Appendix 7. Interpretation I2


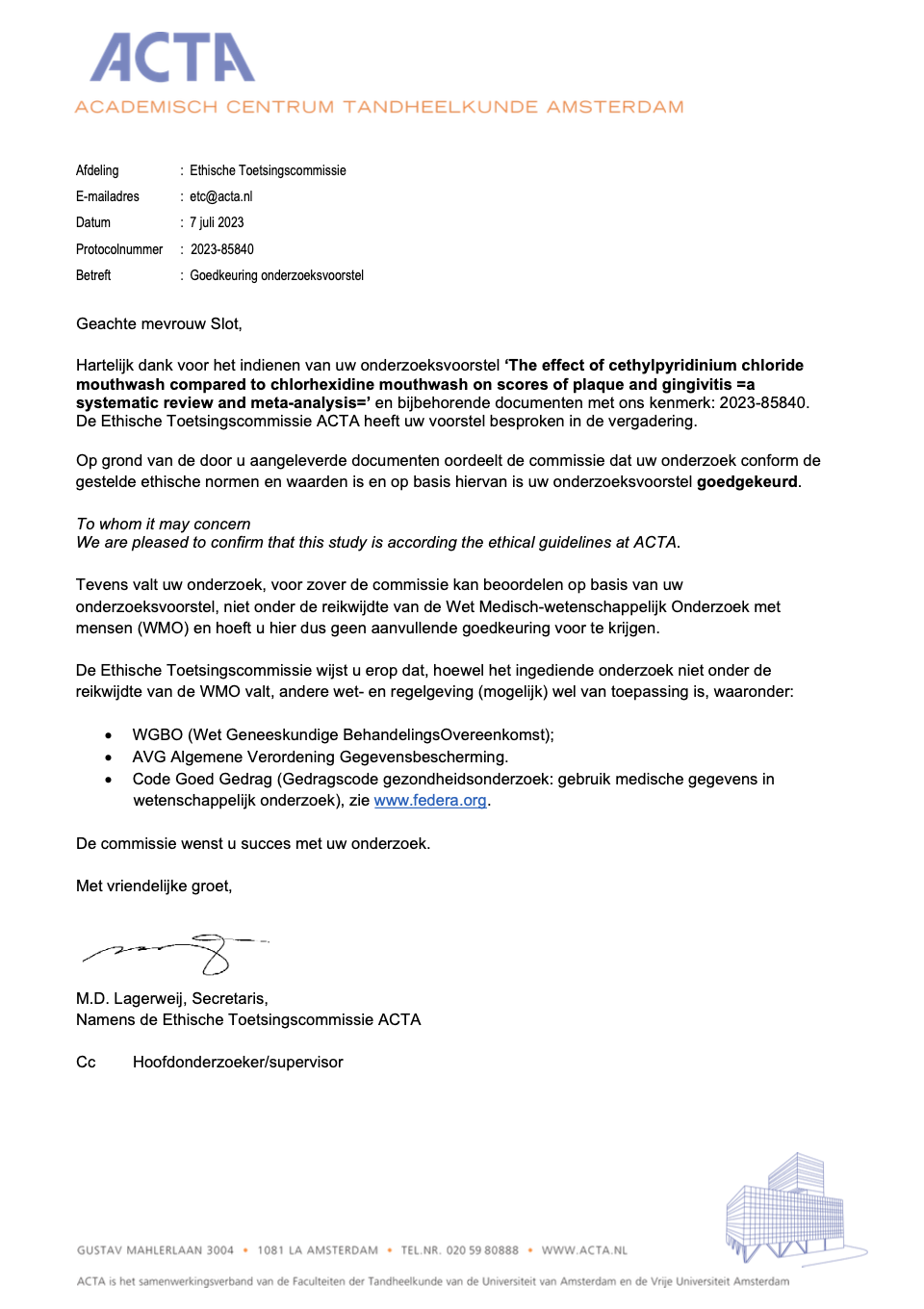
**Appendix 1.** Ethical approval of the ACTA institute

**Appendix 2.** Methodological quality and potential risk of bias scores of the individual studies that were included for this review

| Type of brushing model | Study | Random sequence generation (selection bias) | Allocation concealment  (selection  bias) | Blinding of participants or personnel (performance bias) | Blinding of outcome assessment (detection bias) | Complete outcome data  (attrition bias) | A-selective reporting  (reporting bias) | No other issues | **Overall Risk of Bias** |
| --- | --- | --- | --- | --- | --- | --- | --- | --- | --- |
| **Non-brushing** | **Binney et al. (1992)** (46) | Y | U | U | U | Y | Y | Y | High |
|  | **Jenkins et al. (1994)** (34) | Y | U | Y | Y | Y | Y | Y | Moderate |
|  | **Renton- Harper et al. (1996)** (39) | Y | U | Y | U | Y | Y | Y | High |
|  | **Moran et al. (2000)** (41) | Y | U | N | Y | Y | Y | Y | High |
|  | **Yates et al. (2002)** (40) | Y | U | N | Y | U | Y | Y | High |
|  | **Carvalho et al. (2004)** (36) | Y | U | U | U | Y | Y | Y | High |
|  | **Pizzo et al.**  **(2006)** (37) | Y | U | N | Y | Y | Y | Y | High |
|  | **Rahman et al. (2014)** (43) | Y | U | Y | Y | Y | Y | Y | Moderate |
|  | **Tarlattinia et al. (2018)** (47) | Y | U | U | U | Y | Y | Y | High |
| **Brushing** | **Stookey et al. (2005)** (35) | Y | U | Y | U | U | Y | Y | High |
|  | **Junior et al. (2015)** (42) | Y | U | U | Y | Y | Y | Y | High |
|  | **Miley et al. (2019)** (45) | Y | U | Y | Y | U | Y | Y | High |
|  | **Tadakamadla et al.**  **(2020)** (38) | Y | Y | Y | Y | Y | Y | Y | Low |
|  | **Oo, et al (2023)** (44) | Y | U | Y | Y | Y | Y | Y | Moderate |
|  | 14 studies  Percentage Yes | 100% | 2.4% | 50% | 64.2 % | 78.6% | 100% | 100% | 10x High (71.4%)  3x Moderate (21.4%)  1x Low(7.1%) |

NA: not applicable, PY: probably yes, Y: yes, N: no, U: unclear

**Appendix 3a.** Plaque scores (PS)

Mean (SD) scores for the different intervention groups with various indices and their modifications.

| **Type of brushing model** | **#** | **Index** | **Intervention groups** | **Mean (SD)** | | |
| --- | --- | --- | --- | --- | --- | --- |
|  |  |  | **Product** | **Baseline** | **End** | **Difference** |
| **Non-brushing** | **I.**  **Binney et al. (1992)** | Turesky mod. (1970) Quigley & Hein Plaque (1962) | 0.05% CPC-MW  0.2% CHX-MW | 0 ◊  0 ◊ | 2.22 (0.32)  1.60 (0.31) | +2.22 ◊  +1.60 ◊ |
|  | **II.**  **Jenkins et al. (1994)** | Turesky mod. (1970) Quigley & Hein Plaque (1962) | 0.10% CPC-MW  0.05% CPC-MW  0.12% CHX-MW | 0 ◊  0 ◊  0 ◊ | 1.96 (0.33)  2.13 (0.36)  2.11 (0.50) | +1.96 ◊  +2.13 ◊  +2.11 ◊ |
|  | **III.**  **Renton- Harper et al. (1996)** | Turesky mod. (1970) Quigley & Hein Plaque (1962) | 0.05% CPC-MW  0.12% CHX-MW | 0 ◊  0 ◊ | 2.253 (0.251)  1.709 (0.282) | +2.253 ◊  +1.709 ◊ |
|  | **IV.**  **Moran et al. (2000)** | Turesky mod. (1970) Quigley & Hein Plaque (1962) | 0.05% CPC-MW  0.12% CHX-MW | 0 ◊  0 ◊ | 2.29 (0.53)  1.40 (0.46) | +2.29 ◊  +1.40 ◊ |
|  | **V.**  **Yates et al. (2002)** | Turesky mod. (1970) Quigley & Hein Plaque (1962) | 0.05% CPC-MW  0.2% CHX-MW | ?  ? | ?  ? | +0.56 (?)  -0.05 (?) |
|  | **VI.**  **Carvalho et al. (2004)** (35) | Silness & Löe, plaquel index (1964) | 0.05% CPC-MW  0.2% CHX-MW  0.12% CHX-MW | 0 ◊  0 ◊  0 ◊ | 1.03 (?)  0.72 (?)  0.76 (?) | 1.03 ◊  0.72 ◊  0.76 ◊ |
|  | **VIII.**  **Pizzo et al.**  **(2006)** | Turesky mod. (1970) Quigley & Hein Plaque (1962) | 0.05% CPC-MW  0.2% CHX-MW  0.12% CHX-MW | 0 ◊  0 ◊  0 ◊ | 2.88 (0.6)  1.09 (0.49)  1.41 (0.41) | 2.88 ◊  1.09 ◊  1.41 ◊ |
|  | **IX.**  **Rahman et al. (2014)** | Turesky mod. (1970) Quigley & Hein Plaque (1962) | 0.05% CPC-MW  0.12% CHX-MW | 0 ◊  0 ◊ | 2.58 (0.82)  2.33 (0.66) | +2.58 ◊  +2.33 ◊ |
|  | **XI.**  **Tarlattinia et al. (2018)** | Turesky mod. (1970) Quigley & Hein Plaque (1962) | 0.075% CPC-MW  0.2% CHX-MW | 0  0 | 1.30 (0.35)  1.54 (0.31) | 1.30 (0.35)  1.54 (0.31) |
| **Brushing** | **VII.**  **Stookey et al.**  **(2005)** | Turesky mod. (1970) Quigley & Hein Plaque (1962) | 0.10% CPC-MW  0.075% CPC- MW  0.12% CHX-MW | 2.10 (0.40) ◊  2.15 (0.38) ◊  2.03 (0.39) ◊ | 1.60 (0.46) ◊  1.63 (0.46) ◊  1.35 (0.47) ◊ | -0.50 ◊ (?)  -0.52 ◊ (?)  -0.68 ◊ (?) |
|  | **X.**  **Junior et al. (2015)** | O’Leary et al. (1972) | 0.05% CPC-MW  0.12% CHX-MW | 67.70 (5.67)  69.10 (7.29) | 26.10 (12.27)  13.90 (4.86) | -41.6 (12.27) ◊  -55.2 (4.86) ◊ |
|  | **XII.**  **Miley et al.**  **(2019)** | Turesky mod. (1970) Quigley & Hein Plaque (1962) | 0.05% CPC-MW  0.12% CHX-MW | 1.85 (0.46) ♦  2.05 (0.64) ♦ | 1.55 (0.71) ♦  1.52 (0.77) ♦ | -0.30 (0.46) ♦  -0.54 (0.56) ♦ |
|  | **XIII.**  **Tadakamadla et al.**  **(2020)** | Turesky mod. (1970) Quigley & Hein Plaque (1962) | 0.05% CPC-MW  0.12% CHX-MW | 0.005 (0.01)  0.005 (0.01) | 0.020 (0.02)  0.015 (0.02) | +0.015 (0.02)  +0.010 (0.02) |
|  | **XIV.**  **Oo et al. (2023)** | Turesky mod. (1970) Quigley & Hein Plaque (1962) | 0.05% CPC-MW  0.12% CHX-MW | 0.31 (0.21)  0.27 (0.16) | 0.13 (0.11)  0.09 (0.08) | -0.18 (1.71) ◊  -0.18 (1.67) ◊ |

◊ = calculated by the authors of this review based on the presented data in the selected paper, ♦ = provided by the original author, ? = not Reported/unknown + author

Contacted no response received

**Appendix 3b.** Bleeding scores (BS)

Mean (SD) scores for the different intervention groups with various indices and their modifications.

| **Type of brushing model** | **#** | **Index** | **Intervention groups** | **Mean (SD)** | | |
| --- | --- | --- | --- | --- | --- | --- |
|  |  |  | **Product** | **Baseline** | **End** | **Difference** |
| **Brushing** | **VII.**  **Stookey et al. (2005)** | Silness & Löe, gingival index (1964) | 0.10% CPC-MW  0.075% CPC-MW  0.12% CHX-MW | 19.9 (9.84) ◊  18.6 (8.87) ◊  18.6 (7.28) ◊ | 11.6 (6.17) ◊  11.1 (6.16) ◊  8.8 (6.13) ◊ | -8.3 ◊ (?)  -7.5 ◊ (?)  -9.8 ◊ (?) |
|  | **XII.**  **Miley et al. (2019)** | Silness & Löe, gingival index (1964) | 0.05% CPC-MW  0.12% CHX-MW | 0.62 (0.44) ♦  0.95 (0.60) ♦ | 0.26 (0.35) ♦  0.45 (0.50) ♦ | -0.35 (0.26) ♦  -0.49 (0.43) ♦ |

◊ = calculated by the authors of this review based on the presented data in the selected paper, ♦ = provided by the original author, ? = not Reported/unknown + author contacted

**Appendix 3c.** Gingival Index (GI**)**

Mean (SD) scores for the different intervention groups.

| **Type of brushing model** | **#S** | **Index** | **Intervention groups** | **Mean (SD)** | | |
| --- | --- | --- | --- | --- | --- | --- |
|  |  |  | **Product** | **Baseline** | **End** | **Difference** |
| **Brushing** | **VII.**  **Stookey et al (2005)** | Silness & Löe, gingival index (1964) | 0.10% CPC-MW  0.075% CPC-MW  0.12% CHX-MW | 0.800 (0.183) ◊  0.792 (0.177) ◊  0.794 (0.175) ◊ | 0.548 (0.152) ◊  0.526 (0.154) ◊  0.459 (0.152) ◊ | -0.252 ◊ (?)  -0.266 ◊ (?)  -0.290 ◊ (?) |
|  | **XII.**  **Miley et al. (2019)** | Silness & Löe, gingival index (1964) | 0.05% CPC-MW  0.12% CHX-MW | 0.85 (0.22) ♦  0.93 (0.21) ♦ | 0.61 (0.22) ♦  0.62 (0.23) ♦ | -0.25 (0.14) ♦  -0.31 (0.22) ♦ |
|  | **XIII.**  **Tadakamadla et al.**  **(2020)** | Silness & Löe, gingival index (1964) | 0.05% CPC-MW  0.12% CHX-MW | 0.012 (0.03)  0.005 (0.02) | 0.037 (0.06)  0.038 (0.07) | + 0.025 (0.06)  + 0.033 (0.06) |
|  | **XIV.**  **Oo et al. (2023)** | Silness & Löe, gingival index (1964) | 0.05% CPC-MW  0.12% CHX-MW | 0.12 (0.10)  0.15 (0.15) | 0.02 (0.04)  0.02 (0.04) | -0.1 (0.94) ◊  -0.13 (0.56) ◊ |

◊ = calculated by the authors of this review based on the presented data in the selected paper, ♦ = provided by the original author, ? = not Reported/unknown + author

Contacted

**Appendix 3d.** Stain Index (SI)

Mean (SD) scores for the different intervention groups with various indices and their modifications.

| **Type of brushing model** | **#** | **Index** | **Intervention groups** | **Mean (SD)** | | |
| --- | --- | --- | --- | --- | --- | --- |
|  |  |  | **Product** | **Baseline** | **End** | **Difference** |
| **Brushing** | **XIII.**  **Tadakamadla et al.**  **(2020)** | Lobene Stain Index (1968) | 0.05% CPC-MW  0.12% CHX-MW | 0.88 (0.93)  0.72 (0.89) | 1.04 (1.10)  3.32 (2.98) | + 0.16 (1.46)  + 2.6 (3.0) |
|  | **XIV.**  **Oo et al. (2023)** | Modified Lobene Stain Index (2000) | 0.05% CPC-MW  0.12% CHX-MW | NA  NA | 0.12 (0.34)  0.22 (0.37) | NA  NA |

◊ = calculated by the authors of this review based on the presented data in the selected paper, ♦ = provided by the original author, ? = not Reported/unknown

**Appendix 4: Meta-analyses non-brushing studies.**

**Appendix 4.a.** Forest plot **overall** meta-analysis pooling the **end data** CPC-MW vs CHX-MW for **non-brushing study model** for **plaque index scores**


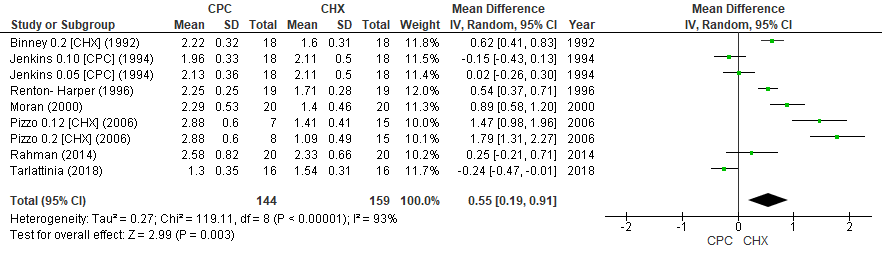


**Appendix 4.b.1.** Forest plot **sub analysis** meta-analysis pooling the **end data** CPC-MW vs CHX-MW for **non-brushing study model** for **plaque index scores, 0.05% CPC-MW vs all concentrations CHX-MW**


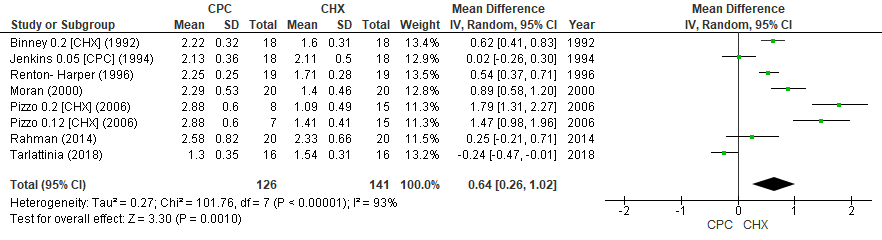


**Appendix 4.b.2.** Forest plot **sub analysis** meta-analysis pooling the **end data** CPC-MW vs CHX-MW for **non-brushing study model** for **plaque index scores, all concentrations CPC-MW vs 0.12% CHX-MW**


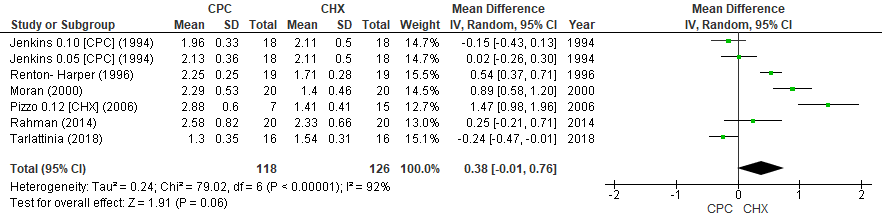


**Appendix 4.b.3.** Forest plot **sub analysis** meta-analysis pooling the **end data** CPC-MW vs CHX-MW for **non-brushing study model** for **plaque index scores, all concentrations CPC-MW vs 0.2% CHX-MW**


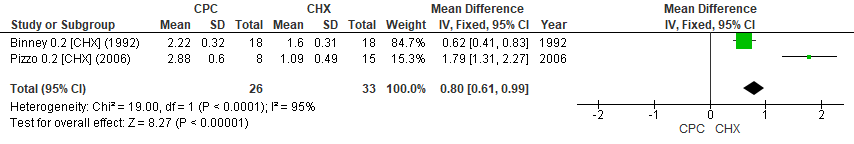


**Appendix 4.b.4.** Forest plot **sub analysis** meta-analysis pooling the **end data** CPC-MW vs CHX-MW for **non-brushing study model** for **plaque index scores, 0.05% CPC-MW vs 0.12% CHX-MW**


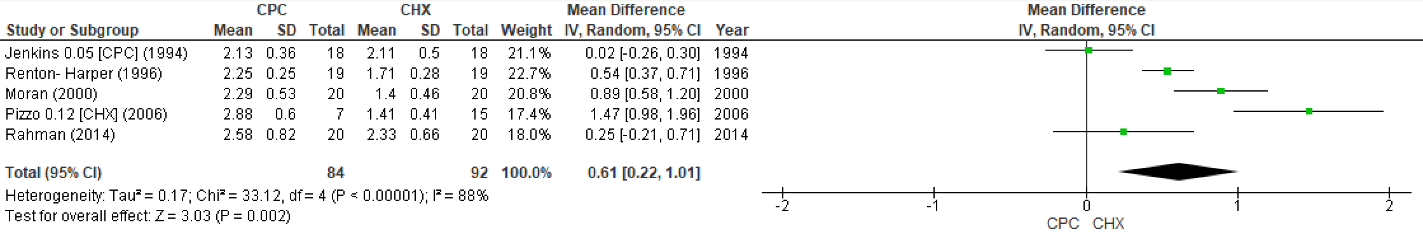


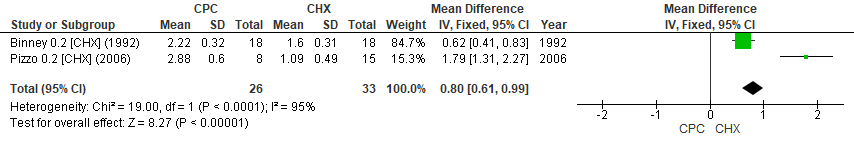
**Appendix 4.b.5.** Forest plot **sub analysis** meta-analysis pooling the **end data** CPC-MW vs CHX-MW for **non-brushing study model** for **plaque index scores, 0.05% CPC-MW vs 0.2% CHX-MW**

**Appendix 5-6: Meta-analyses brushing studies.**

**Appendix 5.a.1.** Forest plot **overall** meta-analysis pooling the **baseline data** CPC-MW vs CHX-MW for **brushing study model** for **plaque index scores**


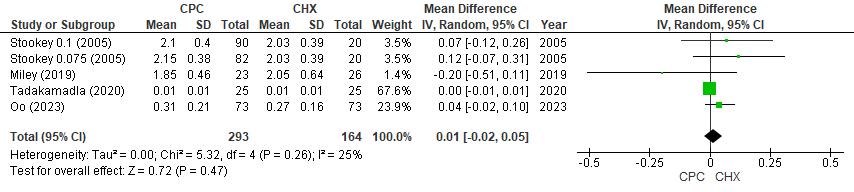


**Appendix 5.a.2** Forest plot **overall** meta-analysis pooling the **end data** CPC-MW vs CHX-MW for **brushing study model** for **plaque index scores**


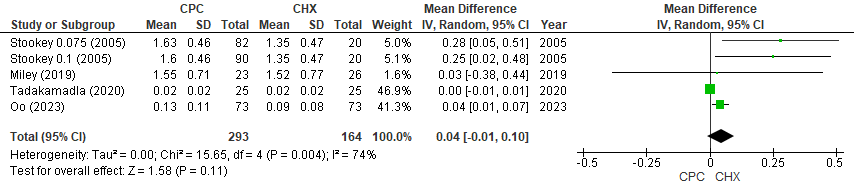


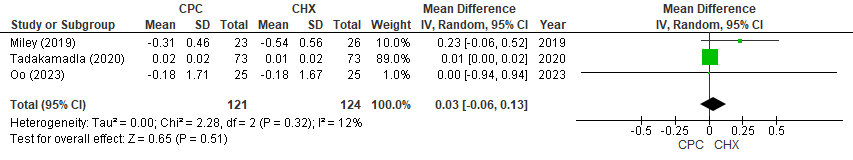
**Appendix 5.a.3.** Forest plot **overall** meta-analysis pooling the **change scores data** CPC-MW vs CHX-MW for **brushing study model** for **plaque index scores**

**Appendix 5.b.1.** Forest plot **sub analysis** meta-analysis pooling the **baseline data** CPC-MW vs CHX-MW for **brushing study model** for **plaque index scores** **0.05% CPC-MW vs 0.12% CHX-MW**


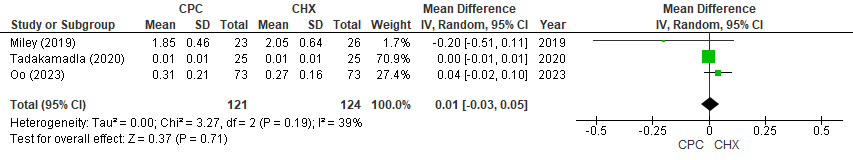


**Appendix 5.b.2.** Forest plot **sub analysis** meta-analysis pooling the **end data** CPC-MW vs CHX-MW for **brushing study model** for **plaque index scores** **0.05% CPC-MW vs 0.12% CHX-MW**


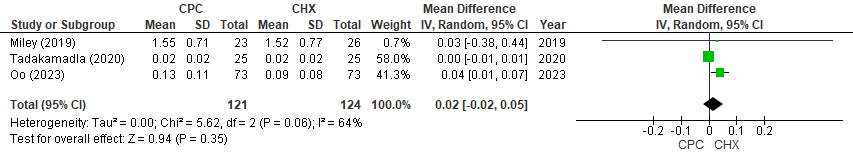


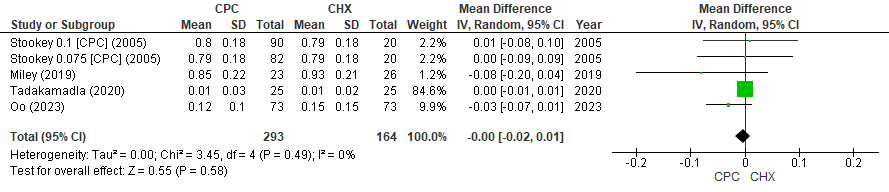
**Appendix 6.a.1.** Forest plot meta-analysis pooling the **baseline data** CPC-MW vs CHX-MW for **brushing study model** for **gingival index scores**


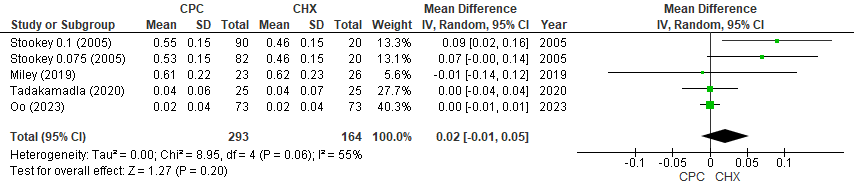
**Appendix 6.a.2.** Forest plot meta-analysis pooling the **end data** CPC-MW vs CHX-MW for **brushing study** **model** for **gingival index scores**


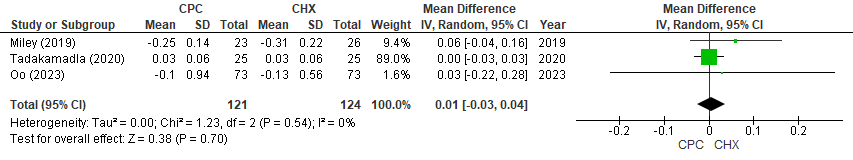
**Appendix 6.a.3** Forest plot meta-analysis pooling the **change scores data** CPC-MW vs CHX-MW for **brushing study model** for **gingival index scores**

**Appendix 6.b.1.** Forest plot **sub analysis** meta-analysis pooling the **baseline data** CPC-MW vs CHX-MW for **brushing study model** for **gingival index scores** **0.05% CPC-MW vs 0.12% CHX-MW**


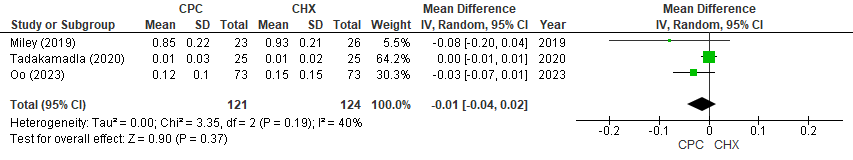


**Appendix 6.b.1.** Forest plot **sub analysis** meta-analysis pooling the **end data** CPC-MW vs CHX-MW for **brushing study model** for **gingival index scores** **0.05% CPC-MW vs 0.12% CHX-MW**
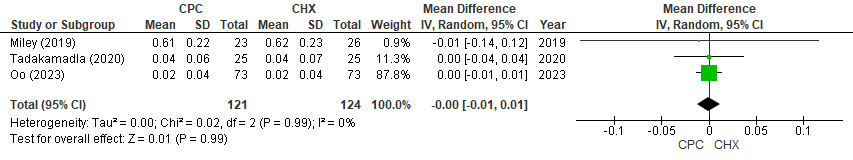


**Appendix 7.** I^2^ value interpretation.(28)

| **Potential not important**  0-40% | **Moderate**  30-60% | **Substantial**  50-90% | **Considerable**  75-100% |
| --- | --- | --- | --- |
